# Using hyperpolarised xenon magnetic resonance imaging to explore breathlessness in long COVID: results from the EXPLAIN study

**DOI:** 10.1183/23120541.00040-2026

**Published:** 2026-09-01

**Authors:** Kher Lik Ng, Laura Saunders, Guilhem Collier, James Grist, Nina Kainth, Victoria Harris, Laurie Smith, Scarlett Strickland, Lotta Gustafsson, Paul Hughes, Ionitiana Evans, Leizl Alvarez, Avianna Laws, Siby Thomas, Alberto Biancardi, Jennifer Rodgers, Samantha Jones, Demi Jakymelen, Martin Brook, Thomas Newman, Megan Plowright, Lily Dryhurst-Pearce, Amany Elbehairy, Kenneth Jacob, Anthony McIntyre, David Capener, Jonathan Bray, Marianne Durrant, Kylie Yeung, Huw Walters, Violet Matthews, Lisa Watson, Gavin Vuddamalay, Gabriele Abu-Eid, Viktorie Madhusudhan Stisova, Mark Cox, Maja Lachut, Nina Mulvey, William Hickes, Alexander Horsley, Helen Davies, Alfred A. Roger Thompson, Fergus Gleeson, Jim Wild, Emily Fraser

**Affiliations:** 1 Division of Clinical Medicine, University of Sheffield, Sheffield, UK; 2 Department of Oncology, University of Oxford, Oxford, UK; 3 POLARIS, University of Sheffield, Sheffield, UK; 4 Department of Physiology, Anatomy and Genetics, University of Oxford, Oxford, UK; 5 Oxford Radiology Research Unit, Oxford University Hospitals NHS Foundation Trust, Oxford, UK; 6 Primary Care Clinical Trials Unit, Nuffield Department of Primary Care Health Sciences, University of Oxford, Oxford, UK; 7 Sheffield Teaching Hospitals NHS Foundation Trust, Sheffield, UK; 8 Department of Respiratory Medicine, Cardiff and Vale University Health Board, Cardiff, UK; 9 Department of Respiratory Medicine, Manchester University NHS Foundation Trust, Manchester, UK; 10 Department of Radiology, Oxford University Hospitals NHS Foundation Trust, Oxford, UK; 11 Department of Radiopharmacy, Oxford University Hospitals NHS Foundation Trust, Oxford, UK; 12 Department of Respiratory Medicine, Oxford University Hospitals NHS Foundation Trust, Oxford, UK; 13 These authors contributed equally to this article as lead authors; 14 These authors contributed equally to this article as senior authors

## Abstract

**Background:**

Breathlessness is a common symptom in long COVID (LC). Using hyperpolarised xenon magnetic resonance imaging (^129^Xe-MRI), we assessed whether this symptom could be attributed to abnormalities in the alveolar–capillary membrane not detected by standard investigations. We focused on never-hospitalised individuals without an identified cause for breathlessness.

**Methods:**

In this prospective, multicentre study, we compared ^129^Xe-MRI, lung function, exercise capacity and symptom questionnaires in LC patients with breathlessness (BLC) to those without breathlessness (NBLC) and healthy controls. Primary outcome was whether BLC demonstrated measurable impairments in gas exchange focusing on dissolved-phase ^129^Xe-MRI metrics: red blood cell to membrane ratio (RBC:M) and red blood cell to gas ratio (RBC:Gas). We also explored associations between symptoms and physiological measures.

**Results:**

Of 269 participants recruited, 196 were included in the analysis (109 BLC, 43 NBLC, 44 controls), with age and sex well matched across groups. BLC had a significantly longer interval from infection to MRI (median 632 days; p<0.001). No significant differences in global or regional RBC:M or RBC:Gas were observed across groups. BLC showed lower forced expiratory volume in 1 s, forced vital capacity, transfer factor of the lung for carbon monoxide (*T*_L__CO_), and carbon monoxide transfer coefficient (*K*_CO_) z-scores compared to controls, though >90% of values remained within normal range. A subset of BLC participants with low *T*_L__CO_ (14 out of 109) showed reduced ^129^Xe-MRI metrics and higher breathlessness scores.

**Interpretation:**

Most nonhospitalised LC participants exhibited no detectable pulmonary abnormalities, including those with breathlessness. However, ∼13% of breathless individuals demonstrated minor reductions in *T*_L__CO_ and ^129^Xe-MRI gas exchange suggesting a potential pulmonary contribution for symptoms in this subgroup.

## Shareable abstract

**Breathlessness is not caused by hidden damage affecting the lungs in the majority of long COVID sufferers. Subtle reductions in gas transfer were observed in 13%. This study highlights the complexity of dyspnoea and importance of rehabilitative approaches.** https://bit.ly/40V5P6U

## Introduction

Post-COVID-19 condition, or long COVID (LC), is estimated to affect two million people living in the UK [1]. It is characterised by a wide range of symptoms, although breathlessness, fatigue and brain fog are most frequently described (Office of National Statistics, April 2024). The mechanisms driving symptoms are complex and, to date, remain poorly understood, but carry a large healthcare burden [2]. Understanding the causes of breathlessness post COVID-19 infection is an important therapeutic priority.

LC is broadly defined by the persistence of symptoms after 12 weeks from the acute infection [3]. The severity of initial symptoms does not predict the development of LC and many patients managed in the community experienced a relatively mild acute COVID-19 illness. Hospital admission is often determined by the requirement for oxygen therapy, and patients typically have evidence of pneumonic changes on lung imaging during the acute phase. These changes can take several months to resolve, and in some cases, result in a fibrotic “footprint” of the acute infection causing persistent lung function impairment and exercise intolerance [4, 5]. In contrast, patients who managed their infection in the community rarely have imaging undertaken during their acute illness, and chest radiograph and computed tomography (CT) to investigate ongoing breathlessness after the infection are normal in most cases [6]. Despite this, up to 50% of LC sufferers report breathlessness, and in some it can be the main symptom limiting the return to functional activity [1].

**FIGURE 1:**
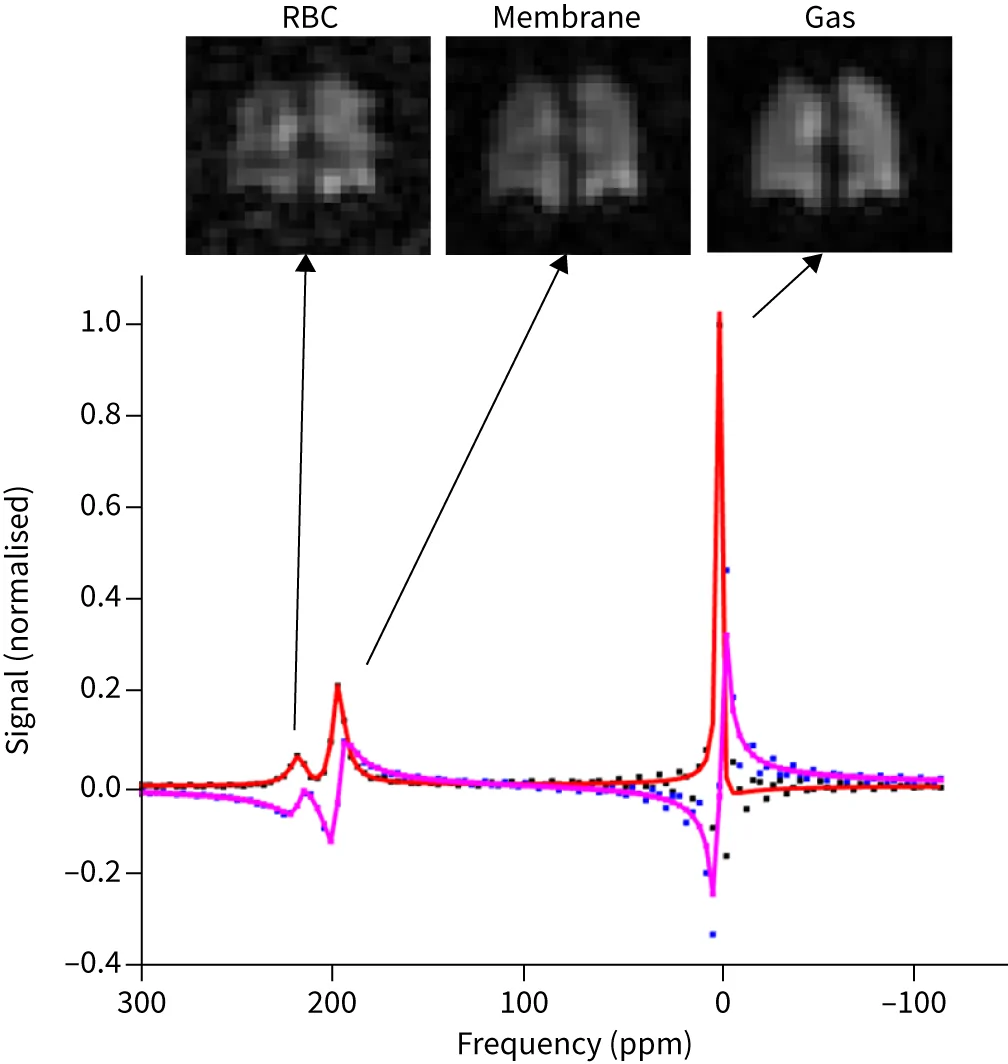
Diagram showing a dissolved phase ^129^Xe spectra. ^129^Xe exhibits a different resonant frequency in the membrane and red blood cells (RBCs) allowing 3D volumetric images of ^129^Xe in the gas, membrane and RBC compartments to be reconstructed. Voxel-wise maps of RBC:Gas, RBC:M and M:Gas are then calculated from the fraction of signal seen in the RBC phase compared to the gas and membrane phase, respectively.

Hyperpolarised xenon magnetic resonance imaging (^129^Xe-MRI) is a novel imaging modality that is safe, reproducible and can provide quantitative metrics [7, 8]. It does not involve radiation exposure. The technique involves inhaling xenon-129 (^129^Xe), an inert gas that has been hyperpolarised to transiently increase signal inside the MRI scanner. The gas mirrors the flow of oxygen through the bronchial tree, across the alveolar–capillary membrane, into the pulmonary vasculature. Delays in the diffusion of gas through the membrane due to abnormalities at this interface, or abnormalities in the pulmonary vasculature, can be quantified with dissolved-phase imaging [9]. After inhalation, ^129^Xe diffuses from the alveoli (gas phase) into the alveolar–capillary membrane. A small percentage of this then diffuses into the blood stream where it transiently binds with haemoglobin in red blood cells (RBCs). ^129^Xe exhibits different resonance frequencies in the gas phase, tissue membrane/plasma phase and on binding within RBCs, and so the ratio of signal delivered to the RBCs compared to the signal measured in the gas or membrane phase can be assessed (figure 1). These RBC:Membrane (RBC:M), Membrane:Gas (M:Gas) and RBC:Gas ratios provide functional measurements of gas exchange at a regional level, providing detailed spatial information about membrane and vascular integrity not necessarily identifiable using global summary measures such as carbon monoxide gas transfer [10]. RBC:M provides a measure of gas transfer efficiency across the membrane and into the capillaries, M:Gas provides a measure of xenon uptake in the membrane and RBC:Gas assesses gas transfer from alveolus to the RBC and therefore provides a broader measurement of the gas exchange properties of the lung.

Previous work has shown impaired ^129^Xe-MRI gas transfer in both hospitalised and nonhospitalised LC patients which fails to normalise over time [11–14]. We reported pilot ^129^Xe-MRI findings of nonhospitalised LC patients infected at the start of the 2020 COVID-19 pandemic with persistent breathlessness and reported significant functional abnormalities [15].

In this study, we again focused on breathlessness, this time in a larger LC cohort. We used ^129^Xe-MRI to help determine whether this key symptom is driven by abnormalities not readily detectable by conventional physiology and imaging tests. We aimed to establish whether abnormalities observed were specific to those with breathlessness or also identified in those with fatigue-predominant presentations without reported breathlessness. Finally, we investigated whether abnormalities observed correlated with lung function tests and measures of dyspnoea and exercise capacity.

## Materials and methods

Participants were diagnosed with LC by a clinical specialist in a dedicated clinic and had symptoms for a minimum of 3 months. With the exception of first-wave participants for whom SARS-CoV2 testing was not readily available, others had a positive PCR or rapid antigen test during their index infection. The symptom of breathlessness was determined at the time of the consultation by the referring physician undertaken at the time of referral to the study. Control participants formed two groups: 1) LC with typical symptoms but without breathlessness (NBLC); 2) participants who had proven COVID-19 infection >3 months previously and had fully recovered. The second control group were recruited by word of mouth and poster advertisement. Written informed consent was obtained from all participants.

Participants who had identifiable explanations for breathlessness were excluded from the study. This included those with CT scores >5 (detailed below), uncontrolled asthma, obstructive spirometry (forced expiratory volume in 1 s (FEV_1_)/forced vital capacity (FVC) <0.7), current smokers, smoking history >10 pack-years and clinically significant cardiorespiratory pathology. Contraindications for ^129^Xe-MRI followed those of conventional MRI: claustrophobia, MRI-incompatible implanted devices or metal and prohibitive body mass index (BMI).

### Study design

This was a prospective, observational, multicentre cohort study enrolling nonhospitalised LC patients from four dedicated post-COVID-19 centres in the UK (Oxford, Sheffield, Cardiff and Manchester) between May 2022 and March 2024. All study procedures were undertaken in Oxford and Sheffield sites and within 6 weeks of ^129^Xe-MRI. This study was designed and implemented with the assistance of a Long COVID Public and Patient Involvement (PPI) group based at the Oxford site. It was approved by the Research Ethics Committee and informed consent was provided by all participants.

Demographic details and comorbidities were collected for all participants. LC participants completed the following patient-reported outcome measures: Dyspnoea-12 (D-12), Functional Assessment of Chronic Illness Therapy–Fatigue (FACIT-F), Generalised Anxiety Disorder 7-item scale (GAD-7) and EuroQoL visual analogue score (VAS).

Pulmonary function tests (PFTs) included FEV_1_, FVC and gas transfer (transfer factor of the lung for carbon monoxide (*T*_L__CO_), alveolar volume (*V*_A_) and carbon monoxide transfer coefficient (*K*_CO_)). Lung function test data are presented as both % predicted and z-scores derived from the European Respiratory Society and Global Lung Function Initiative online calculator [16, 17]. z-scores below 1.64 were considered abnormal. Exertional dyspnoea was assessed by a 1-min sit-to-stand (STS) test, with the number of repetitions recorded alongside modified BORG dyspnoea score (mBORG), heart rate, and oxygen saturations pre- and post-exertion.

The study was funded by the National Institute for Health and Care Research (NIHR) and approved by the South Central Research Ethics Committee (21/SC/0398). It was registered on International Standard Randomised Controlled Trial Number (ISRCTN14304264).

### Imaging protocols

^129^Xe dissolved phase imaging was acquired at either 3T (Oxford) or 1.5T (Sheffield) following inhalation of a 1-L maximum mixture of ^129^Xe and nitrogen from functional residual capacity. Further details are provided in the supplementary material.

Maps of ^129^Xe RBC:M, M:Gas and RBC:Gas were constructed for each subject. Gas transfer ratios acquired at 1.5T and 3T were combined using correction factors based on scanning 14 healthy volunteers at 1.5T and 3T. Age and sex-adjusted z-scores were calculated for each mean metric based on published data [7]. Further details on gas polarisation, image acquisition and post-processing are provided in the supplementary material.

Regional metrics of gas transfer (RBC:M, RBC:Gas) were calculated by dividing whole lung masks evenly in the inferior–superior axis into three regions (lower, middle and upper lungs). Gas transfer maps were also divided evenly in the anterior–posterior axis into two regions corresponding to anterior and posterior lung.

Research CT scanning was performed following 1-L inspiration (for potential comparative purposes with ^129^Xe-MRI) with a slice thickness of 0.625–1.000 mm. Participants who had had a clinical CT to investigate breathlessness did not have a repeat research CT due to the initial scan being unremarkable and additional radiation exposure from a repeat CT scan unjustified. CTs were scored to measure presence of interstitial abnormality, detailed in the supplementary material. The images were reviewed by a senior radiologist blinded to research data.

### Statistical analysis

Analyses were conducted using STATA v18.0. Normality was assessed visually; nonparametric models were used if assumptions were violated. Data are presented as mean±sd (min–max) for normally distributed and median (IQR) (min–max) for non-normally distributed variables. Group comparisons used ANOVA for normally distributed continuous variables, Kruskal–Wallis for non-normal data, and Chi-square or Fisher's exact test for categorical variables.

MRI metrics were summarised by group and compared using linear regression models adjusted for age and sex, unless z-scores already accounted for these. Missing data were not inputed and arose from incomplete questionnaires, inability to perform tests or MRI quality control (due to low image quality, incorrect acquisition of scan parameters or image artefacts).

No multiplicity correction was applied, given the exploratory nature of the analysis. A two-sided p-value <0.05 was considered significant.

The proportion of subjects with ^129^Xe-MRI abnormalities (z-score < −1.64) was summarised by group (breathless and non-breathless *versus* controls) and compared using logistic regression, adjusted for age and sex. Model outputs included group difference estimates, 95% CIs and p-values.

Correlations were assessed between: 1) D-12 and FACIT-F, GAD-7, EuroQoL-VAS; and 2) RBC:Gas, RBC:M and M:Gas z-scores and *T*_L__CO_, *K*_CO_, *V*_A_, spirometry z-scores, BMI and D-12. Pearson or Spearman coefficients were used based on distribution normality.

## Results

### Recruitment

**FIGURE 2:**
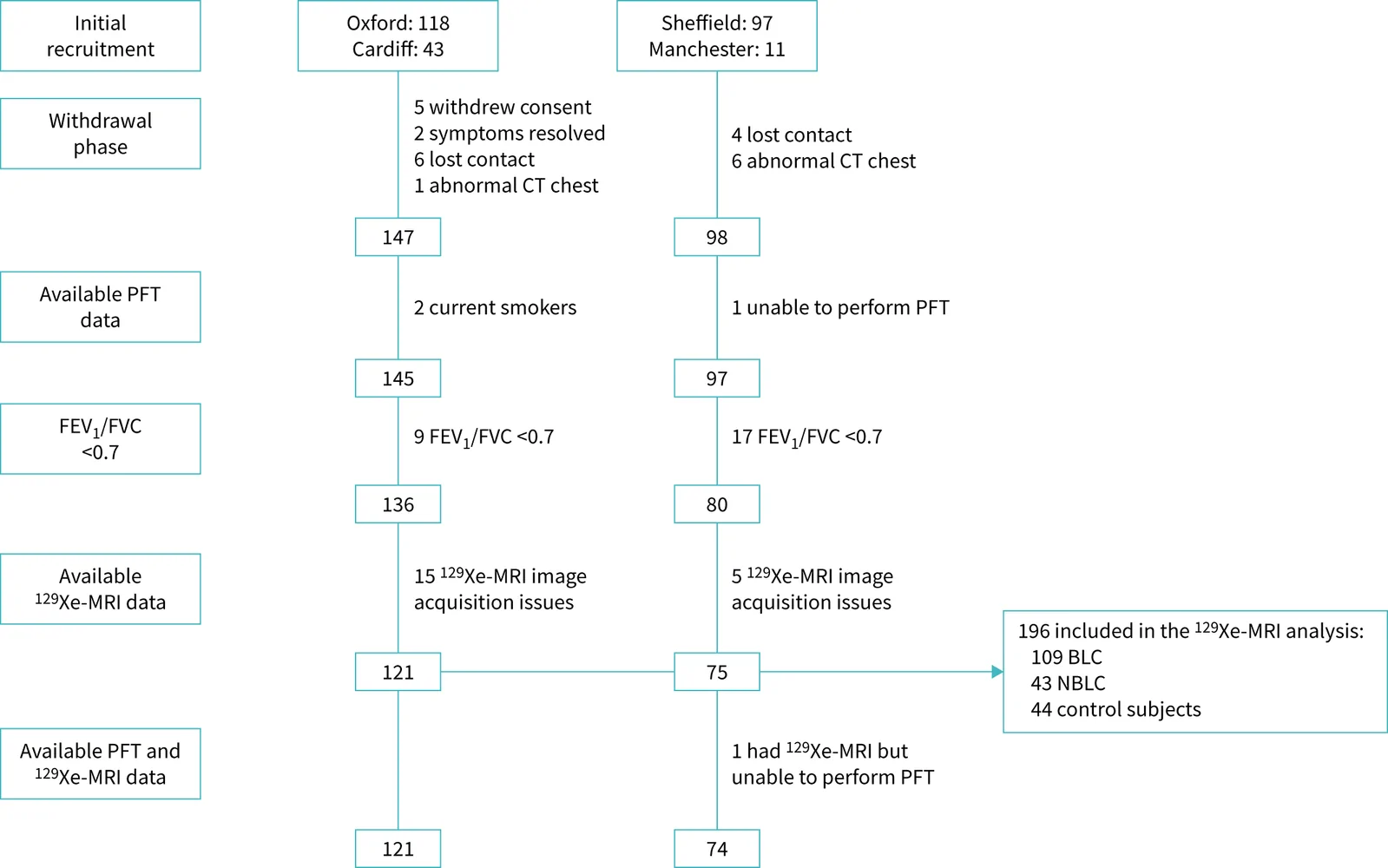
Study profile illustrating participant recruitment, reasons for exclusion and numbers included in analyses. Patients recruited from four centres but all study procedures undertaken in Oxford or Sheffield (Cardiff and Manchester participants, respectively). Patients with obstructive spirometry (FEV_1_/FVC ratio of <0.7) were excluded as this could be an explanation for symptoms. CT: computed tomography; PFT: pulmonary function tests; ^129^Xe-MRI: hyperpolarised xenon MRI; FEV_1_: forced expiratory volume in 1 s; FVC: forced vital capacity; BLC: breathless long COVID; NBLC: non-breathless long COVID.

269 participants were enrolled into the study. 24 were withdrawn due to: withdrawal of consent (five), symptom resolution (two), loss of contact (10) and abnormal CT chest findings (seven). Following ^129^Xe-MRI scan acquisition, 49 were excluded from the final analysis due to: current smoker status (two), obstructive spirometry (FEV_1_/FVC ratio <0.7) (26), inability to complete PFTs (one) and image acquisition/quality issues (20). 196 participants were included in the final ^129^Xe-MRI analysis (109 LC patients with breathlessness (BLC), 43 LC patients who were not breathless (NBLC) and 44 controls) (figure 2).

### Study group characteristics

Age, sex, BMI or past smoking status were not significantly different between breathless long COVID (BLC), non-breathless LC (NBLC) and control participants. There was a longer time period from infection to MRI scanning in the BLC group compared with NBLC and control participants (median 632 days *versus* 352 and 234 days, respectively). The incidence of asthma and diabetes were similar between groups.

No statistically significant differences were found between the BLC and NBLC groups using questionnaires for fatigue (FACIT-F), anxiety (GAD-7) and general health status (EuroQoL-VAS). The dyspnoea was measured with the D-12, which was higher in the BLC than the NBLC group, although there was considerable overlap in the groups and only 13 (30.2%) NBLC participants reported a score of 0 (no breathlessness) (table 1).

**TABLE 1:** The demographics and health characteristics for control, breathless long COVID (LC) and non-breathless long COVID participants included within the study

	Control subjects	Breathless LC	Non-breathless LC	p-value*
**Participants, n**	44	109	43	
**Age, years**	45.7±13.4 (23.3–72.6)	46.9±12.8 (18.8–81.4)	45.9±10.8 (22.1–64.1)	0.821
**BMI, kg** *·* **m^−2^**	28.4±5.8 (19.1–45.5)	28.4±6.4 (16.4–49.5)	27.7±5.1 (20.8–40.0)	0.802
**Ethnicity, n (%)**				p(exact)=0.015
White	38 (86.4%)	94 (86.2%)	43 (100.0%)	
Non-white	6 (13.6%)	15 (13.8%)	0 (0.0%)	
**Sex, n (%)**				p=0.156
Female	34 (77.3%)	71 (65.1%)	25 (58.1%)	
Male	10 (22.7%)	38 (34.9%)	18 (41.9%)	
**CT score^#^**	0 (0–0) (0, 3)	0 (0–0) (0, 4)	0 (0–0) (0, 2)	0.195
Missing, n (%)	0 (0.0%)	1 (0.9%)	0 (0.0%)	
**Time from infection to CT, days^#^**	246 (183–505) (110, 1319)	440 (245–749) (28, 1489)	329 (220–632) (25, 1275)	0.026
Missing, n (%)	0 (0.0%)	2 (1.8%)	0 (0.0%)	
**Time from infection to MRI, days^#^**	234 (180–512) (109, 1319)	632 (349–893) (112, 1489)	352 (212–756) (120, 1275)	<0.001
Missing, n (%)	0 (0.0%)	1 (0.9%)	0 (0.0%)	
**Vaccinated, n (%)**				p(exact)=0.045
No	0 (0.0%)	8 (7.3%)	0 (0.0%)	
Yes	44 (100.0%)	101 (92.7%)	43 (100.0%)	
**Smoking, n (%)**				p=0.729
Former	10 (22.7%)	29 (26.6%)	9 (20.9%)	
**Diabetes, n (%)**				p(exact)=0.797
No	43 (97.7%)	104 (95.4%)	41 (95.3%)	
Yes	1 (2.3%)	5 (4.6%)	2 (4.7%)	
**Asthma, n (%)**				p(exact)=0.386
Childhood only	4 (9.1%)	9 (8.3%)	2 (4.7%)	
No	33 (75.0%)	86 (78.9%)	39 (90.7%)	
**Dyspnoea-12^#^**	NA	13 (9–19) (1, 36)	3 (0–6) (0, 27)	<0.001
**FACIT F**	NA	21±11 (2–52)	23±10 (9–49)	0.413
**GAD-7^#^**	NA	6 (1–11) (0, 21)	5 (3–7) (0, 20)	0.591
**EuroQoL VAS**	NA	53.2±19.1 (0.0–95.0)	55.5±17.4 (20.0–90.0)	0.520
Missing (for questionnaires), n (%)	NA	27 (24.8%)	5 (11.6%)	

Symptom-based questionnaires were evaluated in breathless and non-breathless LC participants only. Data are presented as mean±sd (min–max) unless otherwise stated. Group comparisons based on ANOVA for normally distributed continuous variables, Kruskal–Wallis for non-normally distributed continuous variables, and chi-square or Fisher's exact test for categorical variables. BMI: body mass index; CT: computed tomography; MRI: magnetic resonance imaging; Dyspnoea-12: breathlessness questionnaire; FACIT-F: Functional Assessment of Chronic Illness Therapy-Fatigue; GAD-7: Generalised Anxiety Disorder-7; EuroQoL VAS: EuroQol Visual Analogue Scale “Health Today”. ^#^: median, interquartile range and minimum and maximum presented for non-normally distributed continuous variables.

Combining all LC participants, there was a positive correlation in breathlessness measured by D-12 score with fatigue (FACIT-F), anxiety (GAD-7) and health status (EQ5D-VAS) (supplementary figure S1).

### ^129^Xe-MRI analysis

#### Global analysis

**FIGURE 3:**
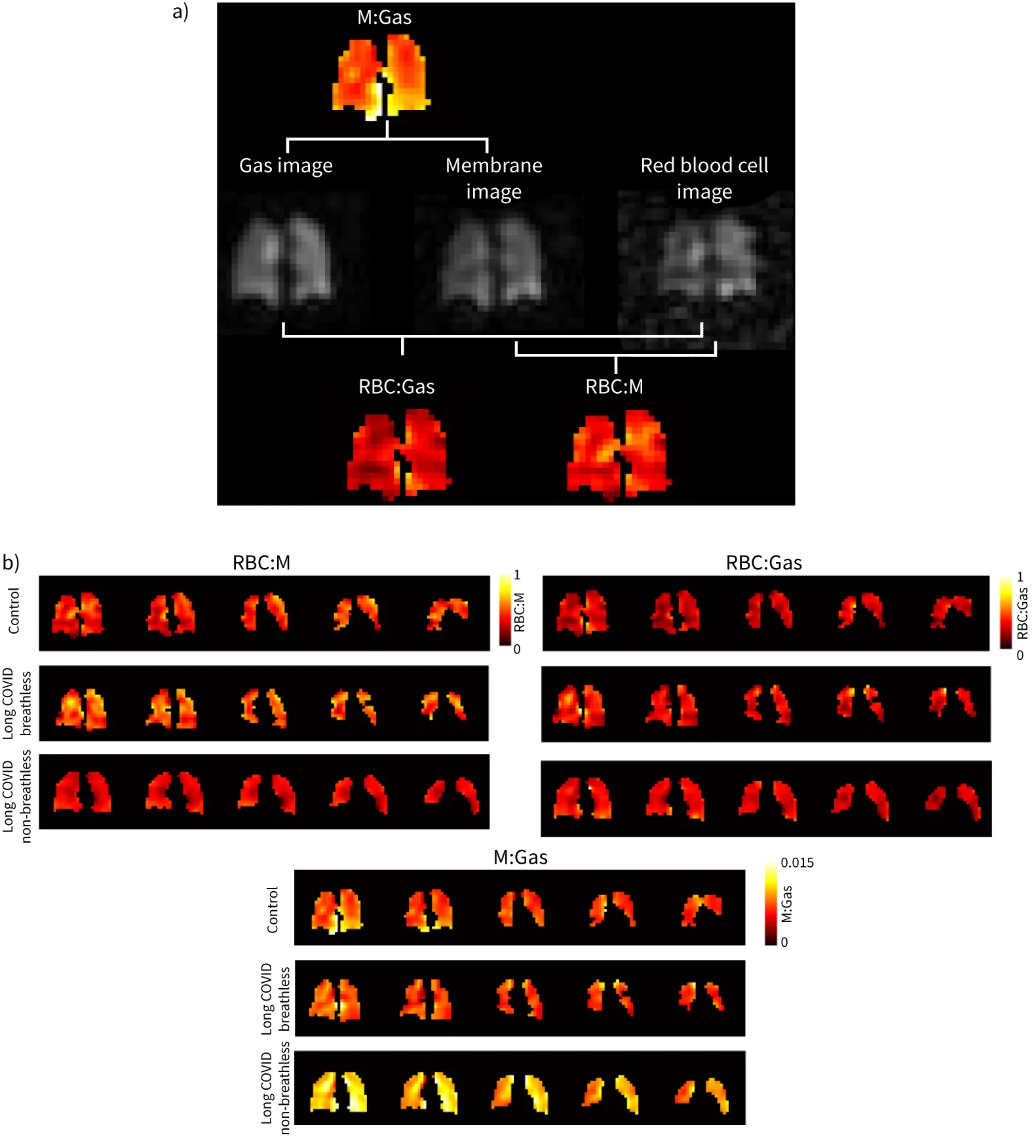
Hyperpolarised xenon pulmonary MRI (^129^Xe-MRI) image maps. a) Figure to demonstrate gas, membrane and red blood cell (RBC) ^129^Xe-MRI image maps and how the ratios of RBC:Gas, RBC:M and M:Gas are calculated from gas and RBC measurements and membrane and RBC uptake, accordingly. b) Representative image maps of the RBC:M, RBC:Gas and M:Gas mean ratio maps of a control (top), breathless long COVID (middle) and non-breathless long COVID (bottom) participant.

**FIGURE 4:**
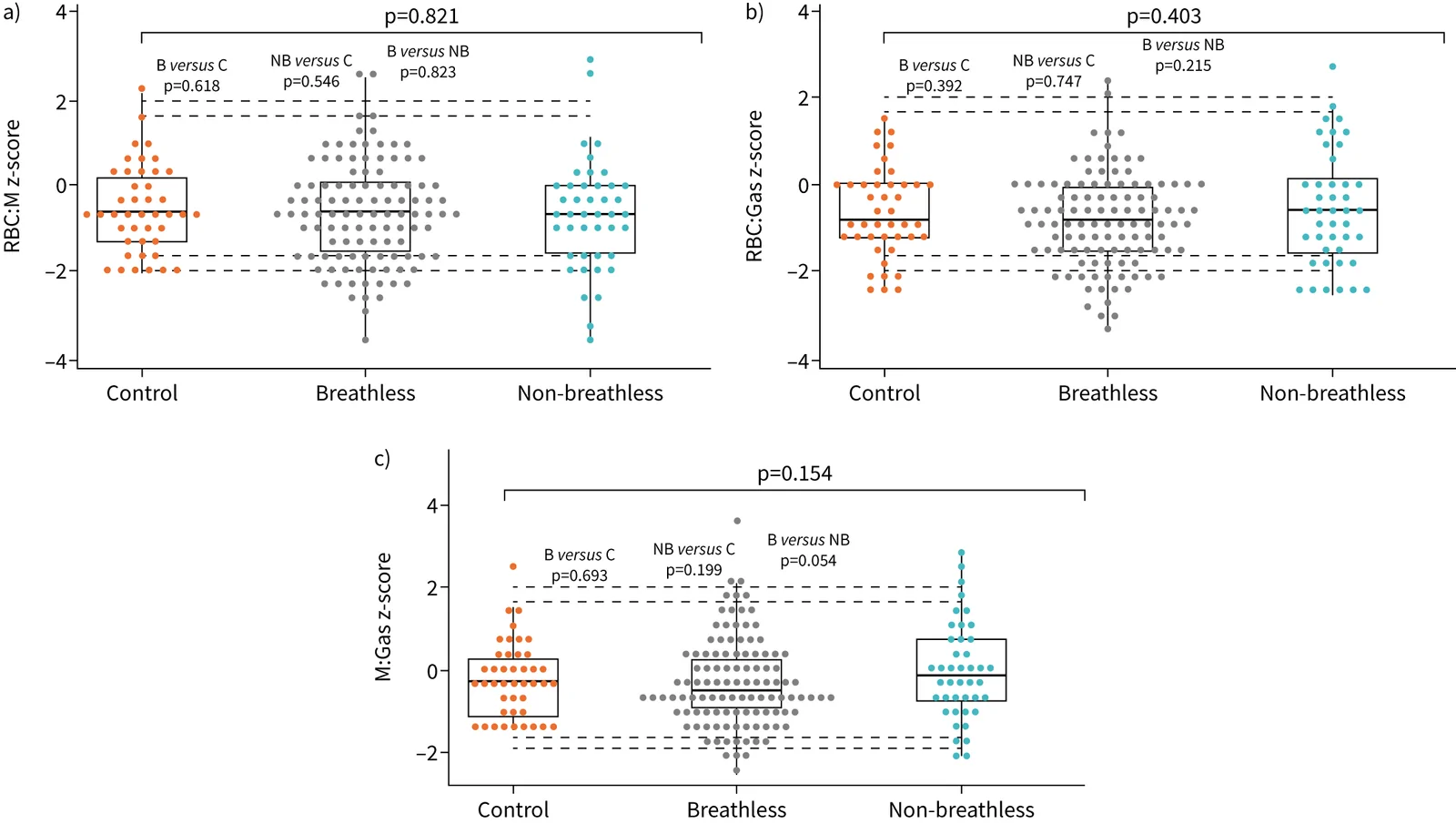
Hyperpolarised xenon pulmonary MRI (^129^Xe-MRI) gas transfer parameters. a) RBC:M, b) RBC:Gas and c) M:Gas z-scores in control (C) subjects, breathless (B) and non-breathless (NB) long COVID groups. ANOVA for overall comparisons between groups, and linear regression for pairwise comparisons. Horizontal dashed lines represent ±1.64 (*i.e.* the 90% normal range, values above 1.64 represent the top 5% and below −1.64 the bottom 5%) and ±1.96 (the 95% normal range, the top and bottom 2.5%).

We analysed global measures of gas exchange using ^129^Xe-MRI. We found no significant differences between BLC, NBLC and control participants in RBC:Gas, RBC:M or M:Gas z-scores (figures 3, 4 and table 2). The percentage of participants with z-scores < −1.64 (“abnormal”) did not differ for either RBC:M z-scores (18%, 19% and 23% for controls, BLC and NBLC, respectively) or RBC:Gas z-scores (16%, 23% and 20%, respectively), although the graphs demonstrated that there was a nonsignificant trend towards lower z-scores in the BLC and NBLC subgroups with a greater proportion having < −1.64 z-scores compared with controls. To assess regional heterogeneity in ^129^Xe distribution, we also measured the coefficient of variation for both RBC:Gas, RBC:M and M:Gas and found no significant differences between groups.

**TABLE 2:** Global hyperpolarised xenon pulmonary MRI (^129^Xe-MRI) measurements in control subjects, breathless and non-breathless long COVID (LC) participants

	Control subjects	Breathless LC	Non-breathless LC	p-value
**Participants, n**	44	109	43	
**RBC:Gas mean**	0.0028±0.0010 (0.0013–0.0053)	0.0027±0.0010 (0.0007–0.0058)	0.0030±0.0012 (0.0012–0.0064)	0.366
**RBC:Gas standard deviation**	0.0010±0.0004 (0.0005–0.0026)	0.0009±0.0003 (0.0003–0.0025)	0.0010±0.0005 (0.0004–0.0025)	0.362
**RBC:Gas coefficient of variation**	0.36±0.09 (0.21–0.64)	0.36±0.12 (0.22–1.07)	0.33±0.06 (0.22–0.46)	0.170
**RBC:Gas z-score**	−0.63±1.00 (−2.41–1.52)	−0.80±1.05 (−3.28–2.37)	−0.55±1.30 (−2.53–2.68)	0.403
**RBC:M mean**	0.35±0.08 (0.23–0.53)	0.35±0.09 (0.16–0.62)	0.36±0.10 (0.19–0.72)	0.824
**RBC:M standard deviation**	0.09±0.03 (0.05–0.18)	0.08±0.02 (0.05–0.17)	0.08±0.02 (0.05–0.17)	0.434
**RBC:M coefficient of variation**	0.27±0.08 (0.12–0.43)	0.26±0.07 (0.14–0.46)	0.24±0.06 (0.13–0.37)	0.153
**RBC:M z-score**	−0.51±1.00 (−2.01–2.15)	−0.62±1.17 (−3.58–2.63)	−0.66±1.30 (−3.47–2.88)	0.821
**M:Gas mean**	0.0085±0.0019 (0.0058–0.0140)	0.0083±0.0023 (0.0035–0.0166)	0.0091±0.0028 (0.0046–0.0184)	0.195
**M:Gas coefficient of variation**	0.2349±0.0635 (0.1464–0.4732)	0.2367±0.0707 (0.1543–0.6640)	0.2224±0.0403 (0.1650–0.3587)	0.264
**M:Gas z-score**	−0.2607±0.9033 (−1.5244–2.3799)	−0.3396±1.0756 (−2.6099–3.6530)	0.0463±1.3399 (−2.1109–4.4811)	0.154
Missing, n (%)	1 (2.3%)	0 (0.0%)	0 (0.0%)	

RBC:Gas, RBC:M and M:Gas z-score and coefficient of variation measurements displayed. Summaries are presented as mean±sd (min–max) unless indicated otherwise. p-values derived from ANOVA, adjusted for age and sex unless already adjusted *via* z-score.

There was a positive correlation between RBC:Gas z-score with laboratory measures of gas transfer, *T*_L__CO_ and *K*_CO_ z-score (r=0.404 and 0.246, respectively, both p<0.001). However, RBC:M z-score was positively correlated with *T*_L__CO_ z-score (r=0.201, p=0.001) but not *K*_CO_ z-score (r=0.089, p=0.103). RBC:M z-score but not RBC:Gas z-score correlated with BMI and *V*_A_. Neither RBC:M and RBC:Gas z-scores were found to correlate with spirometry values or D-12 scores (supplementary table S1).

#### ^129^Xe-MRI regional analysis

To determine if there were regional abnormalities that might be masked by global scores, the lungs of each participant were divided into upper, middle and lower zones and analysed individually (supplementary figure S2). Separately, anterior and posterior lung divisions were also analysed. No significant differences were observed between any of these groups (supplementary table S2).

### Lung function

**FIGURE 5:**
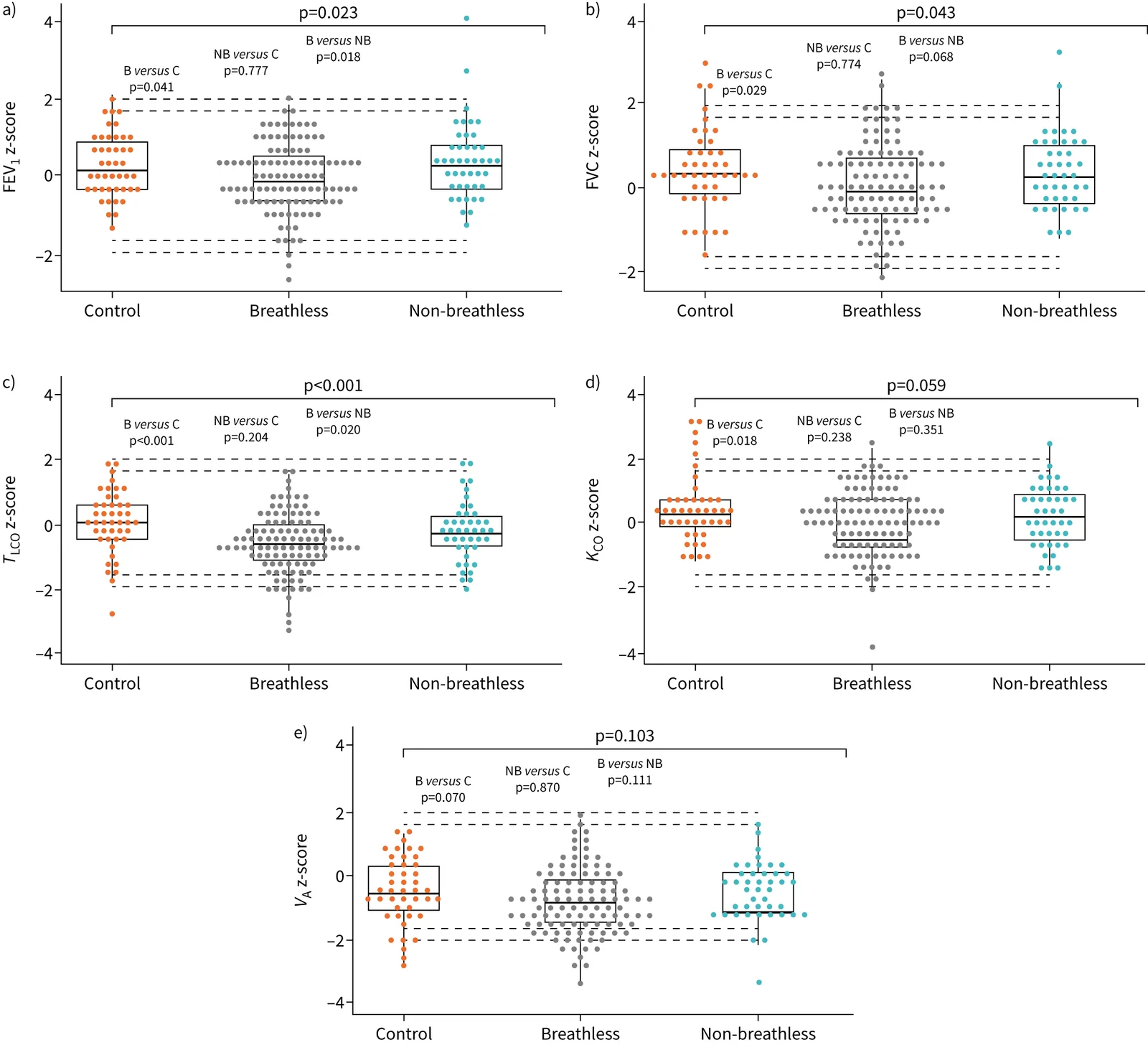
Lung function z-scores presented by group: control subjects (C), breathless (B) and non-breathless (NB) long COVID groups. a) forced expiratory volume in 1 s (FEV_1_), b) forced vital capacity (FVC), c) transfer factor of the lung for carbon monoxide (*T*_L__CO_), d) carbon monoxide transfer coefficient (*K*_CO_) and e) alveolar volume (*V*_A_). Horizontal dashed lines represent ±1.64 (*i.e.* the 90% normal range, values above 1.64 represent the top 5% and below −1.64 the bottom 5%) and ±1.96 (the 95% normal range, the top and bottom 2.5%). p-values are given from an ANOVA model.

The majority of participants had lung function values within normal range, although the group mean z-scores for FEV_1_ and FVC were both statistically lower in the BLC group than the control group (FEV_1_ p=0.023 and FVC p=0.043, respectively, figure 5). Overall, only seven (6.4%) of BLC participants had a z-score < −1.64 (*i.e.* below the lower limit of normal) for FEV_1_ and three (2.7%) had a z-score < −1.64 for FVC.

Group mean *T*_L__CO_ % predicted and z-scores were all within the normal ranges across all groups but were lower in BLC compared to NBLC and controls (BLC 91.2%, NBLC 96.8%, 100.5%; p<0.001 and BLC −0.7, NBLC −0.3, controls 0.0; p<0.001, respectively) (table 3). Mean % predicted *T*_L__CO_ lies within normal range at 91% in the BLC group. The data on lung volume (*V*_A_) demonstrates a similar distribution to the gas transfer values, although in this case, the differences between groups were not statistically significant (figure 5e). The proportion of participants with *T*_L__CO_ z-score < −1.64 was higher in the BLC but not significantly different from the NBLC and control groups (BLC – 14 (13%), NBLC – three (7%), controls – two (4.5%), p=0.279).

**TABLE 3:** Lung function measures by group with tests of group differences.

Measure	Control subjects	Breathless long COVID	Non-breathless long COVID	p-value^#^
**Participants, n**	44	109	43	
**FVC z-score**	0.4±1.0 (−1.5–2.9)	0.0±1.0 (−2.1–2.6)	0.4±0.9 (−1.2–3.3)	**0.043**
**FVC % predicted**	105.4±11.9 (80.4–131.8)	100.8±12.9 (73.7–132.6)	105.1±12.7 (84.8–145.2)	0.053
**FEV_1_ z-score**	0.3±0.8 (−1.2–2.1)	−0.0±0.9 (−2.7–1.9)	0.3±1.0 (−1.3–4.0)	**0.023**
**FEV_1_ % predicted**	103.7±10.2 (82.7–126.5)	99.6±12.0 (65.8–125.3)	104.6±12.8 (84.1–150.6)	**0.0128**
***T*_L__CO_ z-score**	−0.0±1.0 (−2.9–1.7)	−0.7±0.9 (−3.3–1.5)	−0.3±0.9 (−2.1–1.8)	**<0.001**
***T*_L__CO_ % predicted**	100.5±13.5 (66.0–125.2)	91.2±12.8 (58.1–124.4)	96.8±13.3 (73.3–132.9)	**<0.001**
***K*_CO_ z-score**	0.4±1.0 (−1.2–3.3)	0.0±1.0 (−3.7–2.3)	0.2±0.9 (−1.5–2.5)	0.059
***K*_CO_ % predicted**	106.8±15.8 (84.9–149.6)	100.7±13.5 (53.8–131.4)	102.9±13.0 (78.7–136.2)	0.050
***V*_A_ z-score**	−0.5±1.0 (−2.8–1.3)	−0.8±1.0 (−3.5–1.7)	−0.6±0.9 (−3.3–1.5)	0.103
***V*_A_ % predicted**	94.3±11.4 (69.1–115.4)	90.9±11.1 (62.3–122.4)	94.0±10.0 (67.6–118.0)	0.111
Missing, n (%)	0 (0.0%)	1 (0.9%)	0 (0.0%)	

Both z-scores and % predicted values are displayed. Summaries are presented as mean±sd (min–max) unless indicated otherwise. p-values presented in bold indicates statistical significance. FVC: forced vital capacity; FEV_1_: forced expiratory volume in 1 s; *T*_LCO_: transfer factor of the lung for carbon monoxide; *K*_CO_: carbon monoxide transfer coefficient; *V*_A_: alveolar volume. ^#^: p-values derived from ANOVA.

To examine whether the characteristics of BLC participants with lower *T*_L__CO_ z-score (< −1.64, n=14) differed from those with normal values, we compared clinical and physiological data, and found that these participants had higher D-12 scores (mean 22.3 *versus* 13.6), lower *V*_A_ (−1.4 *versus* −0.8, p=0.020) and statistically lower RBC:Gas z-scores (−0.8 *versus* −0.7, p=0.008) and RBC:M z-scores (−1.0 *versus* −0.9, p=0.049). (supplementary table S3).

### Exercise tests and physiological data

Pre-exertion resting mBORG scores were higher in BLC compared to NBLC and controls and remained significantly higher in the BLC participants post-exertion (supplementary figure S3a,b, both p<0.001). Supplementary table S4 demonstrated that BLC participants performed fewer STS repetitions than both NBLC participants and controls (p<0.001 for both absolute number and percentile accounting for age and sex). No differences were seen in oxygen saturations and heart rate pre- and post-STS between the groups.

## Discussion

This is the first large-scale multicentre study to comprehensively investigate whether lung abnormalities are associated with breathlessness in never-hospitalised LC patients. Previous studies using ^129^Xe-MRI in post-COVID-19 and pulmonary fibrosis populations have demonstrated that this modality can detect functional abnormalities at the level of the alveolar–capillary membrane not easily identified with conventional tests. Longitudinal ^129^Xe-MRI studies in other diseases have shown the technique to be highly reproducible and capable of detecting early changes in disease state [18, 19]. In this study, we used ^129^Xe-MRI alongside established respiratory investigations to assess the lungs of breathless and non-breathless LC patients, aiming to understand the relationship between symptoms and lung function and to identify potential underlying pathophysiological processes affecting the lung parenchyma.

Our results show that in the majority of never-hospitalised LC participants, gas transfer measurements using ^129^Xe-MRI were similar to those of fully recovered controls. In a subgroup of breathless LC participants (n=14; 12.9%) with modest reductions in *T*_L__CO_, we found that *T*_L__CO_ correlated with lower RBC:Gas and RBC:M z-scores and higher breathlessness scores, suggesting that a small subset of LC patients may have underlying abnormalities contributing to their symptoms.

In a pilot study, significant reductions in RBC:M scores were observed in 54% (n=6) of breathless LC participants [15]. In contrast, the EXPLAIN study found mild reductions in RBC:M z-scores (<1.64) in only 20% of breathless participants, and no significant difference in the prevalence of RBC:M or RBC:Gas abnormalities between breathless LC participants and controls. These differences may relate to sample size and timing of recruitment [14, 15]. Earlier studies enrolled participants during the initial waves of the pandemic, when infection severity was higher and residual lung damage more likely to be detected on ^129^Xe-MRI. Although EXPLAIN included participants from early waves, most were recruited during later phases, when COVID-19 generally caused milder manifestations [20]. LC participants were diagnosed following evaluation in dedicated clinics according to NICE criteria [3, 21]. Questionnaires related to fatigue, breathlessness and health status confirmed substantial symptom burden, indicating that these findings are likely representative of the broader LC population [1, 6].

Lung function values were generally lower in the predefined breathless LC group compared with non-breathless LC and control participants. However, most z-scores remained within the internationally standardised “normal range”, indicating that observed differences were unlikely to explain the breathlessness reported. While some lung function metrics showed statistically significant differences, these are unlikely to be clinically meaningful or account for symptoms in most breathless LC participants. Pre-COVID lung function was unknown, limiting causal interpretation, although groups were matched for age, sex, BMI, smoking status and comorbidities, reducing potential confounding. Interestingly, lower lung volumes in some LC individuals have also been observed in other nonhospitalised LC cohorts [22].

Comparing breathless participants with *T*_L__CO_ z-scores < −1.64 to those within the normal range revealed some notable differences. Participants with low *T*_L__CO_ had higher D-12 scores, indicating greater breathlessness, and lower RBC:Gas values. Given the heterogeneous pathophysiology of LC, it is plausible that those with low *T*_L__CO_ represent a subgroup in which lung damage contributes to symptoms. These participants also had lower *K*_CO_ and *V*_A_, suggesting that gas transfer abnormalities reflect changes in both ventilated airspace and efficiency of gas exchange between alveoli and pulmonary capillaries [15]. However, caution is warranted in interpreting these findings, as the absolute differences were modest and unlikely to account for the excessive dyspnoea reported. Due to the absence of longitudinal data, it cannot be determined whether impaired gas transfer recovers over time.

The STS test demonstrated that both breathless and non-breathless LC participants performed fewer repetitions than controls, indicating reduced exercise capacity. Deconditioning has been identified as a major factor in exercise limitation among LC populations [23]. The mBORG dyspnoea score, commonly used to quantify breathlessness after exercise, was elevated in about one-third of breathless participants pre-STS [24]. Lung function measures derived from ^129^Xe-MRI and traditional PFTs cannot account for this degree of breathlessness. Alternative/additional explanations include breathing pattern disorder, fatigue, subclinical ventilation abnormalities, deconditioning, anxiety, weight gain and pain [6, 25–27]. Breathlessness and reduced exercise capacity may also relate to metabolic or structural abnormalities outside the lungs. Small studies have linked exercise intolerance to impaired oxygen extraction by skeletal muscle and demonstrated structural and metabolic changes in peripheral muscles of LC participants [28, 29].

This study has limitations. Participants were referred by clinical teams and defined as “breathless” or “non-breathless” following assessment. Breathlessness scores (D-12, mBORG) obtained on the day of research visits showed overlap between groups, with some “non-breathless” participants reporting elevated scores. No correlation was observed between D-12 scores and ^129^Xe-MRI metrics (table 3), highlighting the difficulty of using such a complex and multifaceted symptom to differentiate groups. Several previous studies have also demonstrated poor correlation between breathlessness and objective respiratory pathology [30–32]. A key strength of this study is recruitment from a real-world clinical setting based on self-reported symptoms, reinforcing the finding that the majority of participants do not exhibit lung parenchymal abnormalities explaining their breathlessness.

^129^Xe-MRI is a promising tool for detecting subclinical respiratory disease. The lack of significant differences between groups provides reassurance that lung pathology is not a primary driver of breathlessness for most LC participants. Nevertheless, its use remains largely within research, and its clinical utility and limitations are still emerging. Traditional lung function z-scores are derived from thousands of healthy participants, whereas this study's z-scores were based on 62 volunteers, limiting adjustment for demographic effects on gas transfer metrics [7, 33]. Larger studies are needed to establish normative ^129^Xe-MRI data across age, sex and ethnicity. Additionally, correction factors used to adjust for field-strength differences may not fully account for technical variability, although statistical findings for RBC:M and RBC:Gas were consistent when analysed separately at different field strengths (supplementary tables S5 and S6), supporting the robustness of the conclusions.

In summary, this study did not identify significant gas transfer abnormalities affecting the alveolar–capillary membrane in LC participants. The combination of novel functional imaging and traditional investigations provides evidence that occult lung abnormalities do not account for breathlessness in the majority of LC patients.

## Supplementary material

**Please note:** supplementary material is not edited by the Editorial Office, and is uploaded as it has been supplied by the author. Supplementary material 00040-2026.SUPPLEMENT
