# Supplementary material for: Using hyperpolarised xenon magnetic resonance imaging to explore breathlessness in long COVID: results from the EXPLAIN study
Source: ERJ Open Res. 2026 Sep 1;12(5):00040-2026. doi: 10.1183/23120541.00040-2026 (PMC13531307; doi:10.1183/23120541.00040-2026)
Supplement: Supplementary file 1 [file 00040-2026.SUPPLEMENT.pdf]

Supplementary material

Methods

CT scoring

The following approach was used to visually score CT scans. The presence of ground glass opacification, reticulation, traction bronchial dilatation and honeycombing was assessed and CT images were scored as per the clinical standard of CT COVID-19 severity scoring system.[1] Each lung lobe was assessed individually, and the percentage involvement of the lobe was noted based on visual assessment, scoring 1 if < 5% area involved, 2 if 5–25% area involved, 3 if 25–50% area involved, 4 if 50–75% area involved, and 5 if > 75% area involved. Maximum CT severity score was 25 provided by the sum of the individual lobar scores.

Sit-to-stand test

The number of sit-to-stand repetitions was also reported in percentile (2.5th, 25th, 50th, 75th, 97.5th) using the reference values by age group published by Strassmann et al.[2]

^{129}Xe-MRI

Polarisation of xenon

^{129}Xe gas (86% 129Xe, Spectra Gases Inc.) was polarised as previously described.[3,4] Briefly, ^{129}Xe gas was polarised by rubidium vapour spin exchange optical pumping (SEOP), and cryogenically accumulated in 1-L doses using an in-house polariser (POLARIS, Sheffield) in Sheffield and in Oxford a commercial polariser (Model 9300, Polarean).

A 1L maximum of mixture of ^{129}Xe and nitrogen was inhaled from functional residual capacity.[5] For subjects scanned in Sheffield, the ^{129}Xe dose was titrated for subjects with height <160cm dose.[6] For subjects scanned in Oxford, 1L dose was used for all subjects.

Hyperpolarised xenon MRI Oxford and Sheffield centres

MRI was performed at 1·5T (General Electric Healthcare, WI, SIGNA Artist) in Sheffield and 3T (SIGNA Premier) in Oxford with imaging performed on a dedicated ^{129}Xe thorax transmit receive coil (CMRS in Sheffield and Pulseteq in Oxford) and the ^{1}H body coil (GE Healthcare).

Dissolved phase ^{129}Xe spectroscopic images were acquired using a 3D 4-echo flyback radial acquisition as previously described (image resolution=2·0cm in all dimensions, reconstructed isotropic resolution of 1·25cm, repetition time per spoke = 15ms, flip angle per excitation on dissolved and gas phases = 22 and 0·22 degrees, respectively, total scan time = 14s).[7]

Gas, Membrane (M), and Red Blood Cell (RBC) images were reconstructed, and ratiometric maps (RBC:M, RBC:Gas and M:Gas) were constructed. The mean and coefficient of variation of each map was calculated. Z-scores were calculated for each mean metric based on published data from 62 healthy participants scanned at 1·5 T (including 24 healthy participants from this study).[8] Gas transfer ratios acquired at 1·5T and 3T were combined using correction factors based on scanning 14 healthy volunteers at 1·5 and 3T.

Regional metrics of gas transfer (RBC:M, RBC:Gas, M:Gas) were calculated by dividing whole lung masks evenly in the inferior-superior axis into three regions (lower, middle, and upper lungs). Gas transfer maps were also divided evenly in the anterior-posterior axis into two regions corresponding to anterior and posterior lung.

Results

Correlation between Dyspnoea-12 and patient-reported outcome measures

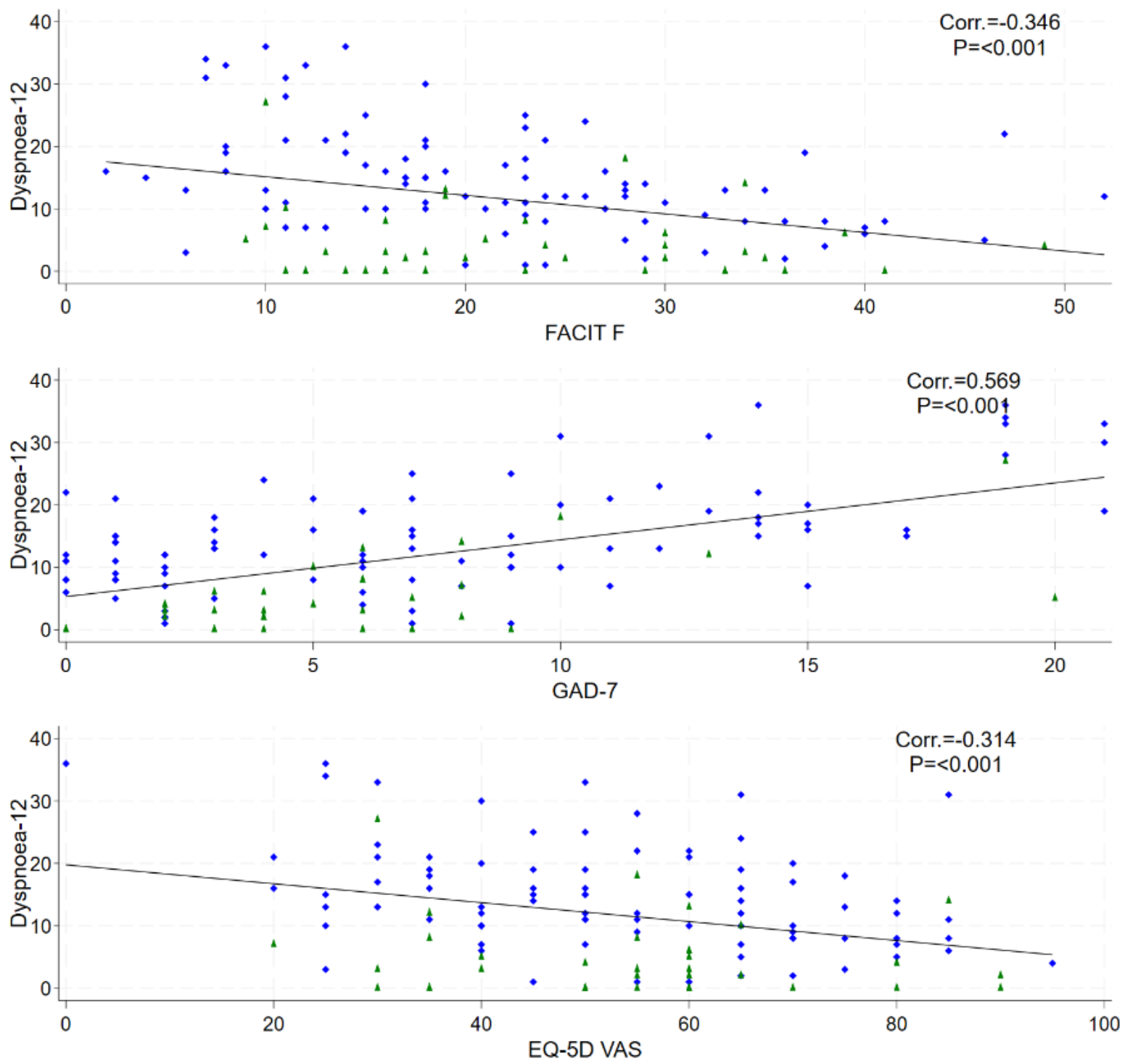

Figure S 1. Graphs showing the correlations between a) FACIT-F b) GAD-7 and c) EQ5D VAS scores and Dyspnoea-12 score in all long COVID (LC) participants. Higher D-12 values indicate higher breathlessness scores, lower FACIT-F scores indicate worse fatigue ratings, increased GAD-7 scores indicate higher anxiety scores, higher EQ-5D VAS scores indicate better perceived health. Blue markers represent the breathless and green the non-breathless LC groups.

FACIT-F – Functional Assessment of Chronic Illness Therapy – Fatigue; GAD-7 – Generalised Anxiety Disorder-7; and EQ-5D VAS – EuroQol Visual Analogue Scale 'Health Today'.

Regional metrics of RBC:Gas z-score

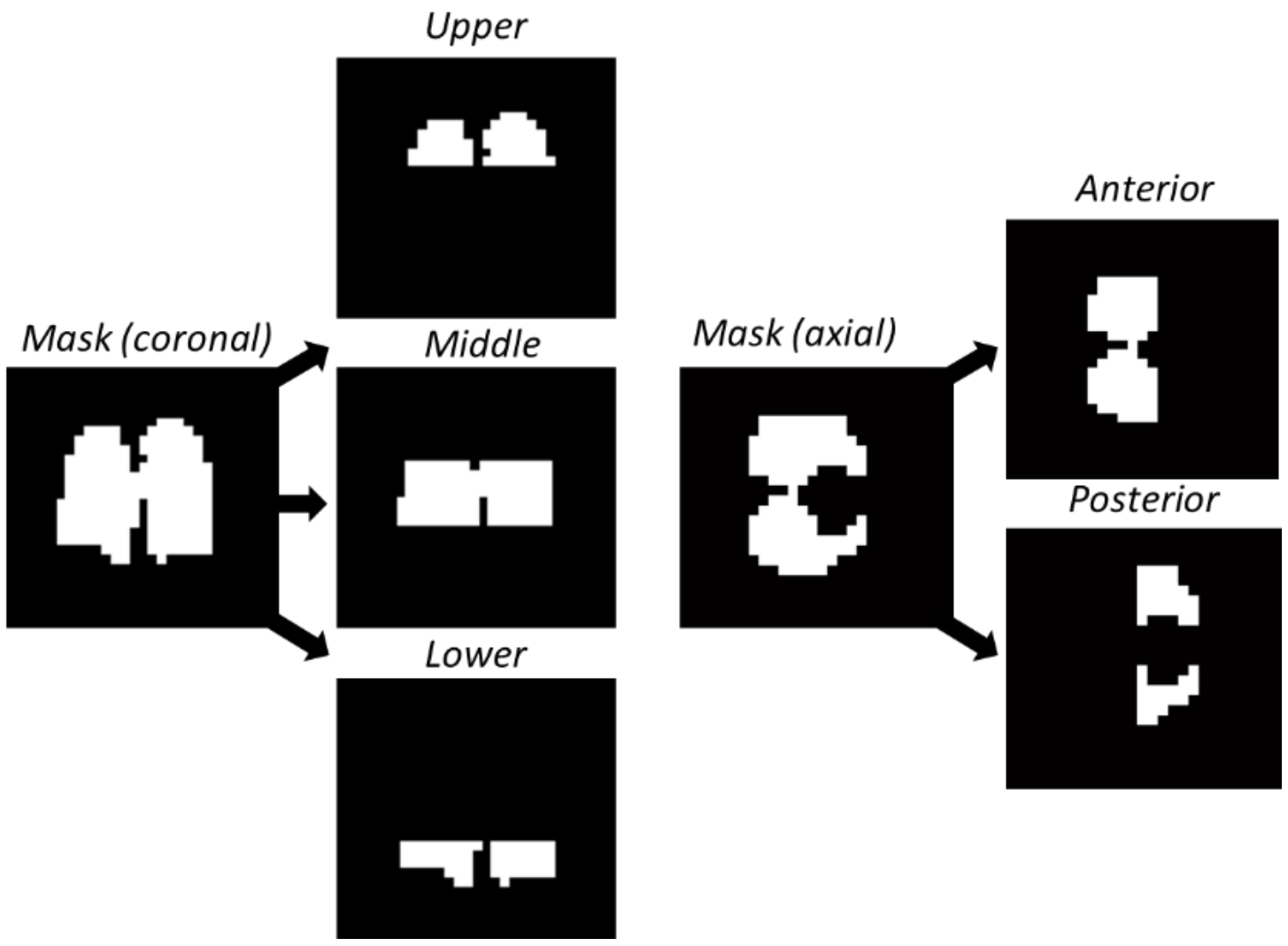

Figure S 2. The lungs were divided into upper, middle and lower areas, as well as anterior and posterior regions. Regional metrics of gas transfer (RBC:M, RBC:Gas and M:Gas z-scores) were calculated within each area to assess for evidence of regional abnormalities.

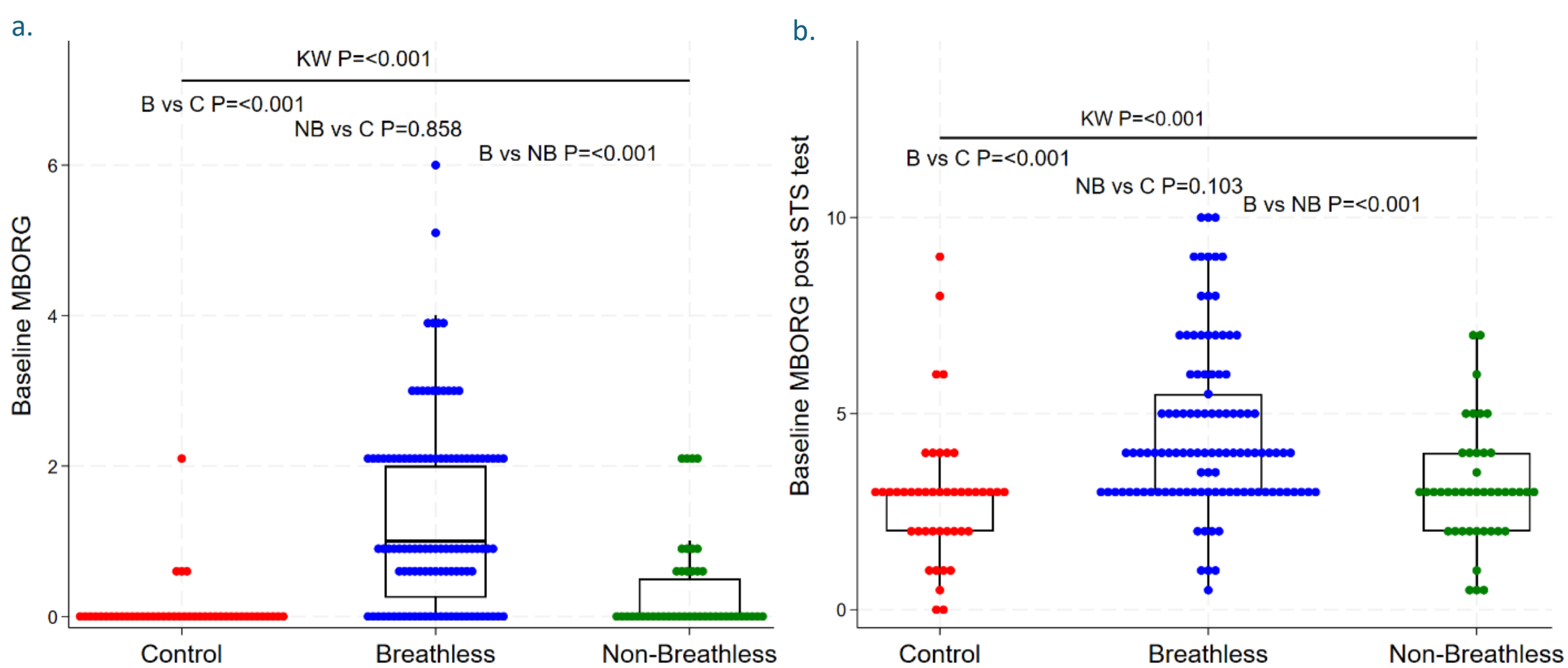

Figure S 3. Comparison of mBORG scores pre and post sit-to-stand test. a) Baseline and b) post STS change in modified BORG Dyspnoea score (mBORG) between controls, breathless (BLC), and non-breathless Long COVID (NBLC) participants.

Variable	Age and sex adjusted Correlation, P-value with RBC:Gas	Age and sex adjusted Correlation, P-value with RBC:M	Age and sex adjusted Correlation, P-value with M:Gas
FEV_1 z-score	0·100, p=0·151	0·040, P=0·467	0·102, P=0·161
FVC z-score	0·062, p=0·373	0·055, P=0·315	0·035, P=0·624
TLCO z-score	**0·304, p<0·001**	**0·185, P=0·001**	0·119, P=0·100
Va z-score	0·080, p=0·249	**0·121, P=0·025**	-0·050, P=0·487
KCO z-score	**0·247, p<0·001**	0·089, P=0·103	**0·171, P=0·018**
BMI	0·002, p=0·978	**-0·128, P=0·025**	**0·185, P=0·014**
Dyspnoea-12	-0·076, p=0·372	-0·050, P=0·455	-0·063, P=0·488

Table S 1: Table to show the relationship between the RBC:Gas, RBC:M and M:Gas with lung function measurements, BMI and the Dyspnoea-12 questionnaire. Correlations estimated from a regression adjusted for age and sex. FEV_1 - Forced Expired Volume in one second; FVC - Forced Vital Capacity; TLCO - Transfer factor for Lung carbon monoxide; KCO - carbon monoxide transfer coefficient; Va - Alveolar Volume, BMI - Body mass index.

RBC:Gas z-score	Controls (N=44)	Breathless Long COVID (N=109)	Non-breathless Long COVID(N=43)	p-value*
Lower z-score	-0·32 (0·90) [-1·88 to 1·98]	-0·48 (0·92) [-2·38 to 2·88]	-0·27 (1·07) [-1·89 to 2·93]	0·40
Mid z-score	-0·71 (0·81) [-2·30 to 0·83]	-0·79 (0·92) [-2·66 to 2·07]	-0·59 (1·10) [-2·43 to 2·62]	0·52
Upper z-score	-0·63 (1·05) [-2·92 to 2·04]	-0·72 (1·08) [-3·36 to 2·58]	-0·47 (1·33) [-2·81 to 3·05]	0·47
Anterior z-score	-0·54 (0·74) [-1·90 to 1·56]	-0·59 (0·76) [-2·14 to 2·10]	-0·48 (0·90) [-2·02 to 1·84]	0·72
Posterior z-score	-0·60 (0·97) [-2·45 to 1·30]	-0·74 (1·01) [-3·16 to 2·42]	-0·48 (1·28) [-2·58 to 2·87]	0·38
Missing, n(%)	1(2·3%)	0 (0·0%)	0 (0·0%)	
RBC:M z-score				
Lower z-score	0·34 (0·08) [0·22 to 0·53]	0·34 (0·08) [0·16 to 0·59]	0·35 (0·10) [0·16 to 0·67]	0·81
Mid z-score	0·35 (0·08) [0·22 to 0·50]	0·35 (0·09) [0·16 to 0·60]	0·35 (0·11) [0·18 to 0·72]	0·94
Upper z-score	0·37 (0·08) [0·22 to 0·58]	0·37 (0·09) [0·18 to 0·66]	0·38 (0·11) [0·20 to 0·78]	0·83
Anterior z-score	0·35 (0·08) [0·20 to 0·52]	0·35 (0·09) [0·17 to 0·60]	0·35 (0·10) [0·19 to 0·67]	0·99
Posterior z-score	0·36 (0·08) [0·25 to 0·54]	0·35 (0·09) [0·16 to 0·63]	0·36 (0·10) [0·18 to 0·75]	0·84
Missing, n(%)	1 (2·3%)	0 (0·0%)	0 (0·0%)	
M:Gas z-score				
Lower z-score	-0·21 (0·86) [-1·43 to 1·47]	-0·30 (1·02) [-2·34 to 3·68]	0·04 (1·17) [-1·90 to 3·32]	0·18
Mid z-score	-0·31 (1·09) [-1·83 to 2·91]	-0·42 (1·30) [-3·20 to 4·68]	0·08 (1·63) [-2·55 to 5·65]	0·12
Upper z-score	-0·27 (0·98) [-1·63 to 3·56]	-0·35 (1·05) [-2·52 to 3·25]	0·02 (1·35) [-2·12 to 4·90]	0·18
Anterior z-score	-0·20 (0·94)	-0·31 (1·02)	0·06 (1·34)	0·16

	[-1·51 to 3·43]	[-2·48 to 4·30]	[-1·91 to 4·92]	
Posterior z-score	-0·28 (0·85)<br>[-1·49 to 1·87]	-0·35 (1·03)<br>[-2·49 to 3·13]	0·05 (1·28)<br>[-2·04 to 4·03]	0·10
Missing, n(%)	1 (2·3%)	0 (0·0%)	0 (0·0%)	

Table S 2: Regional RBC:Gas, RBC:M and M:Gas z-scores in controls, breathless Long COVID and non-breathless Long COVID. RBC:Gas, RBC:M and M:Gas z-scores examined separately in the lower, mid and upper lung zones and anterior and posterior portions of lung. Summaries presented as mean, standard deviation and minimum and maximum. *P-values derived from ANOVA.

Characteristics of breathless patients with TLCO z-score >-1.64 and <-1.64

Outcome	BLC with TLCO >-1·64 (N=94)	BLC with TLCO <-1·64 (N=14)	p-value
FVC z-score	0·1 (0·9)<br>[-2·1 to 2·6]	-0·3 (1·0)<br>[-1·9 to 1·9]	0·20
FEV_1 z-score	-0·0 (0·9)<br>[-2·7 to 1·9]	-0·3 (1·0)<br>[-1·9 to 1·8]	0·33
KCO z-score	0·2 (0·8)<br>[-1·5 to 2·3]	-1·1 (1·1)<br>[-3·7 to 1·4]	**<0·001**
Va z-score	-0·8 (1·0)<br>[-2·9 to 1·7]	-1·4 (1·0)<br>[-3·5 to 0·6]	**0·02**
BMI (kg/m^2)	28·3 (6·0)<br>[17·2 to 49·5]	30·1 (7·9)<br>[16·4 to 40·9]	0·31
Smoking history, n (%)	23 (24·5%)	6 (42·9%)	p(exact) = 0·20
Dyspnoea-12	14 (8)<br>[1 to 36]	22 (10)<br>[2 to 36]	**0·001**
Missing, n (%)	17 (18·1%)	3 (21·4%)	
FACIT F	22 (10)<br>[2 to 52]	18 (18)<br>[7 to 47]	0·34
Missing, n (%)	17 (18·1%)	3 (21·4%)	
GAD-7	6 (6)<br>[0 to 2]	10 (6)<br>[0 to 19]	**0·04**
Missing, n (%)	8 (8·5%)	2 (14·3%)	
Baseline mBORG	1·2 (1·2)<br>[0·0 to 6·0]	1·8 (1·5)<br>[0·0 to 4·0]	0·11
Missing, n (%)	1 (1·1%)	0 (0·0%)	
mBORG post STS test	4·4 (1·9)<br>[1·0 to 10·0]	5·5 (2·8)<br>[0·5 to 10·0]	0·08
Missing, n (%)	2 (2·1%)	0 (0·0%)	
Number STS movements patient completed	27·6 (10·3)<br>[9·0 to 71·0]	22·1 (7·0)<br>[11·0 to 40·0]	0·06
Missing, n (%)	2 (2·1%)	0 (0·0%)	
RBC:Gas z-score	-0·7 (1·0)<br>[-3·3 to 2·4]	-0·8 (1·2)<br>[-2·9 to 2·0]	**0·01**

RBC:M z-score	-0·9 (1·2)<br>[-3·9 to 2·5]	-1·0 (1·2)<br>[-3·9 to 2·5]	**0·049**
M:Gas z-score	-0·3 (1·0)<br>[-2·6 to 2·2]	-0·7 (1·5)<br>[-2·2 to 3·7]	0·16

Table S 3: A comparison of the characteristics of BLC with a TLCO z-score below-1·64 and above -1·64 including lung function values, questionnaires, exercise data, and [129]Xe-MRI findings. NBLC and controls not analysed due to low numbers (n=3 and 2, respectively). Summaries presented as mean, standard deviation and minimum and maximum. P-values derived from ANOVA for between group comparisons. TLCO - Transfer factor for Lung carbon monoxide; FVC - Forced Vital Capacity; FEV_1 - Forced Expired Volume in one second; KCO - carbon monoxide transfer coefficient; Va – Alveolar Volume; BMI – Body mass index; FACIT-F – Functional Assessment of Chronic Illness Therapy – Fatigue; GAD-7 – Generalised Anxiety Disorder-7; STS – 1-minute Sit-to-stand test.

Outcome	*Control subjects (N=44)*	*Breathless Long Covid (N=109)*	*Non-breathless Long Covid (N=43)*	*P-value*[†]
Number STS repetitions	39·2 (13·5)<br>[20 to 68]	26·8 (10·1)<br>[9 to 71]	31·3 (12·2)<br>[8 to 59]	**<0·001**
STS Percentile (n^{th})	25 (2·5 to 56·3)<br>[2·5 to 97·5]	2·5 (2·5 to 2·5)<br>[2·5 to 97·5]	2·5 (2·5 to 25·0)<br>[2·5 to 75·0]	**<0·001**
mBORG pre-STS test	0 (0 to 0)<br>[0 to 2]	1 (0·2 to 2)<br>[0 to 6]	0 (0 to 0·5)<br>[0 to 2]	**<0·001**
mBORG post-STS test[‡]	3 (2 to 3)<br>[0 to 9]	4 (3 to 5·5)<br>[0·5 to 10]	3 (2 to 4)<br>[0·5 to 7]	**<0·001**
Oxygen saturation pre-STS test (%)	98 (1·4)<br>[94 to 100]	97·8 (1·7)<br>[92 to 100]	97·5 (1·7)<br>[94 to 100]	0·420
Oxygen saturation post-STS test (%)	97·4 (1·6)<br>[93 to 100]	97·3 (2·1)<br>[85 to 100]	97·8 (1·3)<br>[95 to 100]	0·247
Heart rate pre-STS test (bpm)	75·4 (13·5)<br>[52 to 112]	78·4 (13·4)<br>[50 to 120]	74·7 (12·2)<br>[54 to 104]	0·207
Heart rate post-STS test (bpm)	100·5 (85·5 to 116)<br>[64 to 152]	101 (91 to 113)<br>[55 to 160]	90 (81 to 115)<br>[56 to 171]	0·368
Missing, n(%)	0 (0%)	2 (1.8%)	0 (0%)	

[‡]Median and interquartile range presented for non-normally distributed continuous variables
[†]ANOVA used for normally distributed variables and Kruskal-Wallis for non-normally distributed variables.

Table S 4: Summary of number of repetitions completed during the 1-minute sit-to-stand test (STS) across groups, plus measures of breathlessness (mBORG), oxygen saturations, and heart rate pre-and post the STS test. Summaries presented as mean, standard deviation and minimum and maximum except for non-normally distributed continuous variables[‡]. mBORG – modified BORG dyspnoea score; bpm – beats per minute.

	Control subjects (N=27)	***Breathless Long COVID (N=66)***	***Non-breathless Long COVID (N=28)***	***P-value****
Age (years)	48·2 (14·8) [27·4 to 72·6]	50·0 (12·6) [23·3 to 81·4]	46·8 (10·0) [28·5 to 63·7]	0·510
BMI (kg/m²)	29·9 (6·3) [19·4 to 45·5]	29·9 (6·6) [16·4 to 49·5]	27·5 (4·6) [20·8 to 37·1]	0·209
Sex, n (%)				p = 0·696
Female	20 (74·1%)	43 (65·2%)	19 (67·9%)	
Male	7 (25·9%)	23 (34·8%)	9 (32·1%)	
Time from infection (days) ‡	418 (206 to 628) [110 to 1319]	574 (347 to 900) [125 to 1489]	298 (195 to 518) [141 to 1064]	**0·004**
Smoking, n (%)				p(exact) = 0·265
Former	6 (22·2%)	20 (30·3%)	4 (14·3%)	
FEV_1 z-score	0·27 (0·79) [-1·21 to 1·67]	-0·04 (0·94) [-2·66 to 1·83]	0·43 (1·04) [-1·28 to 4·02]	0·063
FVC z-score	0·28 (0·95) [-1·48 to 2·86]	-0·02 (0·96) [-2·10 to 2·36]	0·36 (0·94) [-1·20 to 3·26]	0·146
TLCO z-score	-0·31 (0·90) [-2·93 to 1·39]	-0·99 (0·83) [-3·33 to 0·41]	-0·47 (0·88) [-2·09 to 1·23]	**<0·001**
KCO z-score	0.51 (1·09) [-1·16 to 3·10]	-0·17 (1·05) [-3·68 to 1·51]	0·07 (0·98) [-1·51 to 2·46]	**0·020**
Va z-score	-0·96 (0·93) [-2·78 to 1·26]	-1·03 (0·94) [-3·46 to 1·36]	-0·69 (0·94) [-3·25 to 1·51]	0·270
CT score	0·0 (0·0 to 0·0) [0·0 to 3·0]	0·0 (0·0 to 1·0) [0·0 to 4·0]	0·0 (0·0 to 0·0) [0·0 to 2·0]	0·134
RBC:M z-score	-0·67 (0·90) [-2·01 to 1·61]	-0·75 (1·18) [-3·58 to 2·63]	-0·62 (1·24) [-3·21 to 2·68]	0·866
RBC:Gas z-score	-0·84 (1·14) [-2·41 to 1·52]	-1·08 (1·14) [-3·28 to 2·37]	-0·86 (1·27) [-2·53 to 2·68]	0·558
M:Gas z-score	-0·28 (1·06) [-1·52 to 2·38]	-0·49 (1·15) [-2·61 to 3·65]	-0·24 (1·32) [-2·11 to 4·48]	0·552
Missing, n (%) for ^{129}Xe-MRI	1 (3·7%)	0 (0·0%)	0 (0%)	
Dyspnoea-12 ‡	NA	14 (11 to 20) [1 to 36]	3 (0 to 6) [0 to 14]	**<0·001**
Missing, n (%)	NA	8 (12·1%)	3 (10·7%)	

‡ Median, interquartile range and minimum and maximum presented for non-normally distributed continuous variables
†ANOVA used for normally distributed variables and Kruskal-Wallis for non-normally distributed variables.

Table S 5: Demographics, pulmonary function tests, Dyspnoea-12, CT, and global ^{129}Xe-MRI (using 3T scanner) measurements across groups in the Oxford and Cardiff cohort (all study procedures undertaken in Oxford). Summaries presented as mean, standard deviation and minimum and maximum except for non-normally distributed continuous variables‡. BMI – Body mass index; FEV_1 - Forced Expired Volume in one second; FVC - Forced Vital Capacity; TLCO - Transfer factor for Lung carbon monoxide; KCO - carbon monoxide transfer coefficient; Va – Alveolar Volume; CT – Computed Tomography.

	Control subjects (N=17)	Breathless Long COVID (N=43)	Non-breathless Long COVID (N=15)	P-value*
**Age (years)**	41·8 (10·2) [23·3 to 60·5]	42·2 (11·7) [18·8 to 69·1]	44·3 (12·4) [22·1 to 64·1]	0·802
**BMI (kg/m^2)**	26·2 (4·2) [19·1 to 34·4]	26·3 (5·3) [16·6 to 41·5]	28·2 (6·1) [21·1 to 40·0]	0·433
**Sex, n (%)**				**p(exact) = 0·042**
**Female**	14 (82·4%)	28 (65·1%)	6 (40·0%)	
**Male**	3 (17·6%)	15 (34·9%)	9 (60·0%)	
**Time from infection (days)** ‡	183 (172 to 237) [130 to 472]	723 (442 to 910) [189 to 1144]	645 (400 to 892) [235 to 1336]	**<0·001**
Missing, n (%)	0 (0·0%)	2 (4·2%)	0 (0·0%)	
**Smoking, n (%)**				p(exact) = 0·451
**Former**	4 (23·5%)	8 (18·6%)	5 (33·3%)	
**FEV_1 z-score**	0·34 (0·85) [-0·85 to 2·11]	-0·04 (0·86) [-2·39 to 1·89]	0·20 (0·91) [-1·04 to 2·60]	0·297
**FVC z-score**	0·63 (0·94) [-1·04 to 2·36]	0·14 (0·97) [-1·82 to 2·58]	0·35 (0·92) [-0·67 to 0·80]	0·203
**TLCO z-score**	0·49 (0·86) [-1·75 to 1·72]	-0·12 (0·81) [-1·50 to 1·53]	0·18 (0·80) [-0·67 to 1·84]	**0·038**
**KCO z-score**	0·33 (1·00) [-0·90 to 3·29]	0·32 (0·76) [-1·04 to 2·32]	0·42 (0·75) [-0·83 to 1·45]	0·925
**Va z-score**	0·18 (0·78) [-1·58 to 1·30]	-0·53 (1·02) [-2·80 to 1·70]	-0·27 (0·75) [-1·27 to 1·27]	**0·031**
Missing, n (%) for all PFTs	0 (0·0%)	1 (2·3%)	1 (6·7%)	
**CT score**	0·0 (0·0 to 0·0) [0·0 to 0·0]	0·0 (0·0 to 0·0) [0·0 to 1·0]	0·0 (0·0 to 0·0) [0·0 to 0·0]	0·683
Missing, n (%)	0 (0·0%)	1 (2·1%)	0 (0%)	
**RBC:M z-score**	-0·27 (1·12) [-1·95 to 2·15]	-0·41 (1·14) [-2·34 to 2·52]	-0·75 (1·43) [-3·47 to 2·88]	0·511
**RBC:Gas z-score**	-0·30 (0·64) [-1·16 to 1·17]	-0·36 (0·72) [-2·18 to 1·31]	0·03 (1·20) [-1·85 to 1·73]	0·291
**M:Gas z-score**	-0·23 (0·63) [-1·46 to 0·67]	-0·10 (0·92) [-1·79 to 1·90]	0·59 (1·25) [-1·73 to 2·79]	0·030
**Dyspnoea-12**‡	NA	14 (8 to 19) [1 to 28]	4 (0 to 5) [0 to 18]	**0·002**
Missing, n (%)	NA	19 (44·2%)	2 (13·3%)	

‡ Median, interquartile range and minimum and maximum presented for non-normally distributed continuous variables
†ANOVA used for normally distributed variables and Kruskal-Wallis for non-normally distributed variables.

Table S 6: Demographics, pulmonary function tests, Dyspnoea-12, CT, and global ^{129}Xe-MRI (using 1.5T scanner) measurements across groups in the Sheffield and Manchester cohort (all study procedures undertaken in Sheffield). Summaries presented as mean, standard deviation and minimum and maximum except for non-normally distributed continuous variables‡. BMI – Body mass index; FEV_1 - Forced Expired Volume in one second; FVC - Forced Vital Capacity; TLCO - Transfer factor for Lung carbon monoxide; KCO - carbon monoxide transfer coefficient; Va – Alveolar Volume; PFTs – Pulmonary function tests; CT – Computed Tomography.

References

1 Francone M, Iafrate F, Masci GM, et al. Chest CT score in COVID-19 patients: correlation with disease severity and short-term prognosis. *Eur Radiol* 2020; 30(12): 6808–6817.

2 Strassmann A, Steurer-Stey C, Lana KD, et al. Population-based reference values for the 1-min sit-to-stand test. *Int J Public Health* 2013; 58(6): 949–953.

3 Chen M, Doganay O, Matin T, et al. Delayed ventilation assessment using fast dynamic hyperpolarised Xenon-129 magnetic resonance imaging. *Eur Radiol* 2020; 30(2): 1145–1155.

4 Norquay G, Collier GJ, Rodgers OI, et al. Standalone portable xenon-129 hyperpolariser for multicentre clinical magnetic resonance imaging of the lungs. *Br J Radiol* 2022; 95(1132): 20210872.

5 Saunders LC, Collier GJ, Chan HF, et al. Longitudinal Lung Function Assessment of Patients Hospitalized With COVID-19 Using (1)H and (129)Xe Lung MRI. *Chest* 2023; 164(3): 700–716.

6 Smith LJ, Horsley A, Bray J, et al. The assessment of short- and long-term changes in lung function in cystic fibrosis using ^{129}Xe MRI. *Eur Respir J* 2020; 56(6): 2000441.

7 Collier GJ, Eaden JA, Hughes PJ, et al. Dissolved 129Xe lung MRI with four-echo 3D radial spectroscopic imaging: quantification of regional gas transfer in idiopathic pulmonary fibrosis. *Magn Reson Med* 2021; 85(5): 2622–2633.

8 Collier GJ, Smith LJ, Saunders LC, et al. Age, sex, and lung volume dependence of dissolved xenon-129 MRI gas exchange metrics. *Magn Reson Med* 2024; 92(4): 1471–1483.
